# The Longitudinal Association Between Social Factors, Edentulism, and Cluster of Behaviors

**DOI:** 10.3390/geriatrics10060142

**Published:** 2025-10-31

**Authors:** Fatimah Alobaidi, Ellie Heidari, Wael Sabbah

**Affiliations:** Faculty of Dentistry, Oral & Craniofacial Sciences, King’s College London, London SE5 9RS, UK

**Keywords:** cluster analysis, dentition, health behavior, latent class analysis, structural equation modeling, social class

## Abstract

**Objective:** This study aimed to explore the direct relationships between social determinants and behavioral clusters, as well as their potential indirect associations mediated by edentulism. **Methods:** Information on social variables (collected in Wave 3, 2006/07), edentulism (Wave 5, 2010/11), and health-related behaviors (Wave 7, 2014/15) was drawn from the English Longitudinal Study of Ageing (ELSA). Baseline sociodemographic characteristics, including age, gender, ethnicity, education, and wealth, were accounted for. Latent class analysis (LCA) was applied to four behavioral indicators—smoking status, alcohol consumption, fruit and vegetable intake, and physical activity—to identify behavioral clusters. A confirmatory factor analysis (CFA) was then used to construct a latent variable representing social support and social networks. Two structural equation models (SEM) were developed to examine both the direct associations between social support/network and behavioral clusters, and the indirect associations mediated by edentulism. **Results:** In LCA, the two-class model was the best fit for the data. Class 1 (risky behaviors) had 7%, while Class 2 (healthy behaviors) had 93%. In SEM Model 1, higher social support/network levels predicted being in the healthy cluster directly (SC = 0.147) and indirectly (SC = 0.009). In Model 2, accounting for wealth and education, higher levels of social support/network maintained the direct association with the healthy cluster (SC = 0.132), but the indirect path lost significance. **Conclusions:** This study found that greater social support was associated with healthier behaviors, and this relationship may be mediated by edentulism. Health policies that encourage social interaction could therefore improve both general and oral health.

## 1. Introduction

Health-risky behaviors are defined as any harmful actions that increase the likelihood of diseases or delay healing [1]. These behaviors often do not occur in isolation but instead cluster together [2,3]. For instance, a study found that two or more risky behaviors were clustered among 68% of adults in England [4]. Substantial evidence has examined the influence of socioeconomic conditions on the clustering of behaviors, including smoking, alcohol use, and unhealthy diet, showing that lower socioeconomic status is associated with a higher likelihood of engaging in multiple risky behaviors [3,5]. This highlights that co-occurring behaviors pose greater health risks and that their distribution is heavily influenced by social relationships and structural inequalities.

Health inequalities arise from the concentration of people with risky behaviors in lower social status. Those individuals are more likely to engage in harmful behaviors such as smoking, high sugar intake, low fruit and vegetable consumption, and poor oral hygiene, all of which are linked to an increased risk of oral diseases [6,7]. Social relationships, particularly social support and social networks have been recognized as important determinants of health [8]. Social environments (social networks and connections), material living conditions (financial difficulties and poor housing), and psychosocial factors (stress and social support) have been shown to shape health behaviors [9,10,11]. In the context of oral health, inadequate social support has been linked to an increased risk of edentulism [12,13], suggesting that weak social ties may translate into poorer oral health outcomes.

Edentulism was selected as a potential mediator in this study because tooth loss represents more than a biological endpoint. It can influence diet, reducing intake of fruit and vegetables and increasing reliance on softer, often less healthy foods [14], thereby directly shaping health behaviors. Beyond functional limitations, tooth loss carries psychosocial consequences, including lower self-esteem, social withdrawal, and loneliness [15]. These psychosocial consequences may, in turn, elevate the risk of engaging in unhealthy behaviors such as smoking, excessive alcohol use, and physical inactivity [8,16]. While other oral health indicators are also important, edentulism may act as a more integrative marker of the cumulative impact of social and behavioral disadvantages across the life course [17]. Tooth loss reflects not only biological and behavioral pathways but also the psychosocial burden associated with impaired oral function and aesthetics [18,19]. Thus, it provides a plausible pathway through which social factors could influence clustering of health-risky behaviors.

Some studies have linked social factors to behavioral clustering [12,13]; however, no known study has investigated the mediating role of edentulism between social support, social networks, and cluster of behaviors. Therefore, the aim of this study was to investigate the direct association between social factors and clusters of behaviors and their indirect association through edentulism. We hypothesize that higher social support and network increase the likelihood of being dentate and engagement in healthy behaviors.

## 2. Material and Methods

### 2.1. Study Design

Data were obtained from the English Longitudinal Study of Ageing (ELSA), specifically from Wave 3 (2006/07), Wave 5 (2010/11), and Wave 7 (2014/15). ELSA is an ongoing, nationally representative study initiated in 2002, comprising more than 12,000 adults aged 50 years and older living in private households across England [20]. The cohort was originally derived from participants in the Health Survey for England (HSE) conducted in 1998, 1999, and 2001. The sampling design follows a multistage stratified random approach, where strata are defined and participants are randomly chosen through computer-assisted selection to ensure representativeness of the older English population. Ethical approval for each wave of ELSA was granted by the Multicentre Research and Ethics Committee (reference: MREC/01/2/91).

Data collection involved computer-assisted personal interviews, supplemented by a self-completed questionnaire, administered by trained interviewers following ELSA protocols. Reliability was ensured through interviewer training, pilot testing, built-in data validation checks, and rigorous quality control and data cleaning procedure. The survey collected data on health-related behaviors such as smoking, alcohol use, fruit and vegetable consumption, and physical activity, along with information on social support and social networks. Wave 3 was selected as the baseline since it was the first to include measurements of all four behaviors.

### 2.2. Measurements

Four health-related behaviors were extracted from Wave 7 to create the outcome cluster of behavior and the baseline at Wave 3, including smoking, alcohol consumption, fruit and vegetable intake, and physical activity. Each behavior was treated as a dichotomous variable, with cut-off points defined in line with previous studies. Smoking (smokers vs. non-smokers) and alcohol intake (≤14 units per week vs. >14 units) reflected UK recommendations for low-risk drinking [21]; fruit and vegetable consumption (<5 portions per day vs. ≥5 portions) followed the WHO guidelines [22]; and physical activity (no or low activity per week vs. moderate or high activity) was based on established criteria for older adults [23].

Social support, the primary exposure variable, was derived from Wave 3 and assessed using questions about participants’ relationships with their spouse or partner, children, friends, and other relatives. Positive social support was captured using three items that asked how much participants felt understood by, could rely on, and could confide in these individuals. Negative social support was measured using three parallel items assessing the extent to which others were perceived as critical, disappointing, or anxiety-provoking. Responses to these six questions were rated on a four-point Likert scale, and scores were summed to yield a total ranging from 0 to 64. Social network size was assessed based on the number of close ties with children, family members, and friends. Emotional closeness with a spouse was also rated on a scale from 0 (“not at all”) to 3 (“a lot”), and all items were combined to generate a composite score ranging from 0 to 28 [24].

Edentulism was collected in Wave 5 and was self-reported based on the presence or absence of natural teeth. The participants were asked, “In relation to your dental health, which of the following applies to you?”. The response options were as follows: (1) no natural teeth and wear dentures; (2) both natural teeth and denture(s); (3) only natural teeth; or (4) neither natural teeth nor dentures. The variable was categorized as edentate (1 and 4) versus dentate (2 and 3). Baseline demographics such as gender, ethnicity, and age (in Wave 3 and Wave 5) were also included. Due to data availability and the need to protect participant identities, several ethnic groups were removed from the dataset. As a result, ethnicity was categorized as white vs. multi-ethnic, consistent with how it is collected in ELSA.

### 2.3. Statistical Analysis

To identify the most appropriate number of behavioral clusters, model fit was evaluated using several statistical criteria, including the Akaike Information Criterion (AIC), Bayesian Information Criterion (BIC), and the Adjusted Bayesian Information Criterion (A-BIC) [25,26], with lower scores indicating superior model fit. A confirmatory factor analysis (CFA) was performed to validate the measurement structure of the latent construct representing social support and social networks. Subsequently, structural equation modeling (SEM) was applied to investigate the direct pathways between social support/networks and behavioral clusters, as well as the indirect pathways mediated by edentulism. The initial model was controlled for demographic factors and baseline behavioral clusters, while a second model additionally accounted for socioeconomic indicators, namely education and wealth. Also, the latent variable and the mediator were regressed on wealth to improve the fit of the model. Model fit was evaluated using the Tucker–Lewis index (TLI), comparative fit index (CFI), and root mean square error of approximation (RMSEA). A good model fit was defined as RMSEA < 0.05 and CFI/TLI > 0.90 [27]. A sensitivity analysis was conducted using fruit and vegetable consumption as an alternative outcome to the cluster of behaviors to examine whether the association between social support and oral health remained consistent when focusing on a single behavior. This approach helps to determine whether the observed relationships hold when analyzing a specific component of the behavioral cluster that has the strongest bidirectional association with oral health outcomes. A secondary sensitivity analysis was also performed to explore the interaction between social support or network and socioeconomic factors (education and wealth) using logistic regression. This was done to assess whether the relationship between social support and behavioral outcomes varied across levels of socioeconomic position.

## 3. Results

### 3.1. Sample Characteristics

A total of 4402 participants had complete data at Wave 3 (Supplementary Figure S1). Of them, 2287 had complete follow-up data at Wave 5 and 7 and were therefore included in the analysis. Table 1 presents the characteristics of the included and excluded sample. In the included sample, 51% were female and 99% were white, with a mean age of 62.2, and most participants had higher levels of education (44%). The mean wealth was 3.6, social support was 45.1, and social network was 9.3. At Wave 5, most included samples had some natural teeth (92%) with a mean age of 66.3. At Wave 7, the majority of participants were non-smokers (93%), consumed 14 or less alcoholic drinks per week (87%), had less than five portions of fruits and vegetables per day (83%), and had moderate or high levels of physical activity (83%). There was a significant difference between the included and excluded samples in terms of sociodemographic characteristics, behaviors, and oral outcomes, suggesting that the study findings may underestimate the results.

### 3.2. Latent Class Analysis (LCA)

One to four classes were generated to test the best class option for the latent class analysis (Supplementary Table S1). Based on the model fit indices, the two-class solution provided the best fit for the data. Most participants (93%) were classified into Class 2 (Healthy), while only 7% were assigned to Class 1 (Risky). Table 2 shows the estimated probabilities of health-related behaviors for each class. Individuals in the risky cluster were unlikely to consume more than five portions of fruits and vegetables (2%), had a high likelihood of smoking (66%), a high likelihood of engaging in moderate or high physical activity (68%), and a moderate likelihood of consuming over 14 units of alcohol per week (20%) compared with the healthy cluster. Conversely, participants in the healthy cluster had a moderate likelihood of eating more than five portions of fruits and vegetables (18%), the lowest likelihood of smoking (2%), the highest likelihood of engaging in moderate or high physical activity (84%), and the lowest likelihood of consuming more than 14 units of alcohol per week (13%) compared with the risky cluster. Demographically, the risky cluster was predominantly male and had lower education levels, whereas the healthy cluster had equal distribution of gender with higher education levels. In both clusters, the majority of participants were white and dentate. The average age was 59.3 years in the risky cluster and 62.4 years in the healthy cluster. Both groups reported relatively high scores for social support, moderate wealth scores, and lower social network across the two groups.

### 3.3. Structure Equation Modeling (SEM)

**Figure 1:**
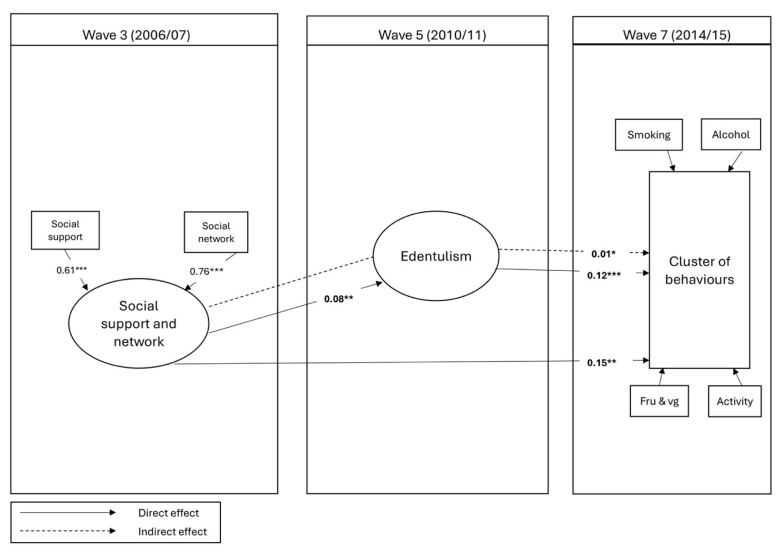
SEM Model 1 for the association between social support and network and cluster of behaviors through edentulism. * *p* < 0.001; ** *p* < 0.01; *** *p* < 0.05.

Table 3 shows the CFA and SEM model, which includes the latent variable (social support and network) and reports standardized coefficients for direct, indirect, and total effects among social support and network, edentulism, and behavioral clusters (Figure 1). The factor loadings for the CFA were significant (*p* < 0.001), with SC = 0.61 (95% CI: 0.423, 0.800) for social support and SC = 0.75 (95% CI: 0.517, 0.988) for social network, indicating satisfactory construct reliability. The model demonstrated a good fit and was adjusted for baseline demographics, baseline cluster of behaviors, and age at Wave 5. The item loadings for the latent variable were significant. Social support and network at Wave 3 were directly associated with edentulism at Wave 5 (SC = 0.08). Furthermore, higher social support and network levels directly (SC = 0.15) and indirectly (SC = 0.01) predicted being in the healthy cluster at Wave 7. Additionally, being dentate at Wave 5 was a direct predictor of belonging to the healthier cluster at Wave 7 (SC: 0.12).

Table 4 presents the CFA and SEM models, incorporating socioeconomic factors (education and wealth). In this model, the association between social support and network at Wave 3 and edentulism at Wave 5 was no longer significant. However, higher levels of social support and network remained a direct predictor of being in the healthy cluster (SC = 0.13), though the indirect path was no longer significant. Additionally, being dentate at Wave 5 predicted belonging to the healthy cluster at Wave 7. Lastly, higher wealth at Wave 3 predicted higher social support and network at Wave 3 (SC = 0.14) and being dentate at Wave 5 (SC = 0.16).

### 3.4. Sensitivity Analyses

A sensitivity analysis using fruit and vegetable consumption instead of cluster of behaviors was conducted. The results remained consistent with the main analysis, showing that higher levels of social support and being dentate were associated with increased fruit and vegetable consumption (Supplementary Table S2). A secondary sensitivity analysis was conducted to explore the interaction between social support or network and socioeconomic factors (Supplementary Table S3). A logistic regression showed that the interaction between wealth and social support was statistically significant (OR = 1.03, 95% CI: 1.01–1.04, *p* = 0.001), indicating that the protective effect of social support on health behaviors was stronger among individuals with higher wealth. In contrast, the interaction between education and social support was not significant. Consistent with the main model, tooth loss at Wave 5 and belonging to the risky behavioral cluster at Wave 3 were both significant predictors of poorer behavioral outcomes at Wave 7.

## 4. Discussion

This study examined the relationship between social support and social network, and behavioral clusters directly as well as indirectly through edentulism. The results indicate that social support and social networks are important factors in shaping health behavior. There was a longitudinal relationship between social support and social networks and cluster of behaviors, and the relationship could be mediated by edentulism. However, socioeconomic characteristics were considered, highlighting that the “explanatory” role of edentulism is limited when SES is accounted for. This suggests that wealth and education act as more fundamental determinants that shape both social resources and oral health across the course of life.

The finding that social support and networks are linked to clusters of health-related behaviors aligns with previous research showing that risky behaviors tend to cluster within populations and are strongly shaped by social and economic conditions [3,4]. Individuals with lower socioeconomic status were more likely to engage in multiple risky behaviors, such as smoking, excessive alcohol consumption, and poor diet, simultaneously [5]. Social support and networks are essential components of an individual’s social environment, influencing health behaviors through emotional, informational, and practical support [28,29]. When these social resources are limited, as often seen in lower socioeconomic groups, individuals may be less motivated to adopt or maintain healthy behaviors [5]. Therefore, the current results support the hypothesis that social connectedness plays a protective role, while its absence can increase the likelihood of engaging in multiple health-risk behaviors, reinforcing patterns already observed in populations facing socioeconomic disadvantage.

Nevertheless, the weakening of the indirect effect after adjustment for socioeconomic factors highlights the importance of structural determinants. Wealth and education may enhance opportunities for healthier lifestyles by increasing access to stable housing, healthy foods, and preventive care [30,31], while also enabling stronger social participation and networks [32]. In this way, socioeconomic resources may serve as upstream conditions that shape both oral health trajectories (such as risk of edentulism) and behavioral clustering, thereby reducing the explanatory power of social support and networks alone. This emphasizes that while edentulism may influence health behaviors, its mediating effect is overshadowed by socioeconomic factors, indicating that only moderate causal inference can be drawn from this pathway.

Unlike previous research suggesting a direct association between social relationships and oral health outcomes [12], the current results indicate that this relationship weakens when socioeconomic factors are included in the model. This aligns with previous suggestions that emphasized the importance of financial and educational resources in determining long-term health outcomes [10]. Furthermore, the findings revealed that higher wealth was significantly associated with both greater social support and network and being dentate. This highlights the important role that socioeconomic status plays in shaping both social and oral health outcomes. Wealthier individuals may have better access to social opportunities, community participation, and stable living conditions, all of which contribute to stronger social networks and perceived support [9,33]. Furthermore, wealth may directly influence oral health by facilitating access to regular dental visits, preventive care, and healthier lifestyles, ultimately reducing the risk of edentulism [34].

Furthermore, edentulism may influence people’s engagement in various health-related behaviors, having a significant effect beyond oral health. Several studies suggested that being edentate may make it more difficult to maintain a healthy lifestyle, by limiting social engagement [15,35] and dietary options [36], two factors that are known to influence health-related behaviors [14,37]. Reduced consumption of fruits and vegetables as a result of masticatory function impairment is one of the most frequent effects of edentulism [38]. Due to their inability to chew fibrous or crunchy foods, such as raw fruits and vegetables, people without teeth are increasingly choosing softer, processed, and frequently less nutrient-dense options [39]. These dietary compromises may not only undermine overall health but may also increase the likelihood of engagement in other risky health behaviors. For instance, these dietary patterns cluster with other unhealthy behaviors, like smoking or physical inactivity [3,4], which often cluster together and are particularly evident among socioeconomically disadvantaged individuals. It is possible that in a similar way, tooth loss may contribute to clusters of behaviors by constraining health-promoting behaviors and possibly reaffirming health risks. Similarly, edentulism may have psychosocial implications that may be related. Tooth loss is often cited as a source of embarrassment or low self-esteem and may restrict social participation [37,40], potentially transforming into increased social isolation and loneliness, both known risk factors contributing to multiple harmful behaviors, like smoking, alcohol intake, and poor dietary behaviors [41,42].

This study has a few limitations that should be discussed. First, the use of self-reported data raises the possibility of recall bias. However, this type of data has conventional legitimacy in epidemiological studies. Second, substantial missing data may have affected the representativeness of the analytic sample. The excluded participants were more likely to come from disadvantaged backgrounds, which raises concerns about selection bias. This attrition may limit the generalizability of the findings. Therefore, the strength of associations may have been underestimated, and the magnitude of data loss remains an important limitation that should be acknowledged when interpreting the results. Third, the dataset from ELSA contains limited detail on the number of tooth loss, restricting the analysis to the use of complete tooth loss. Finally, exploring ethnic differences was not possible because this information was intentionally withheld for ethical reasons.

The research highlights the role of social support and social networks in promoting healthier lifestyles, particularly with older adults. Clinically, these findings suggest that strengthening social connections could support oral health interventions, including prosthetic rehabilitation and preventive care, by encouraging engagement and adherence to treatment. From a policy perspective, integrating social support strategies into community-based and primary care programs may help reduce oral health inequalities, improve access to dental care, and foster healthier behaviors among older adults. Policies addressing broader socioeconomic disparities (e.g., education and financial support) are also likely to enhance the impact of these interventions. Future research should explore longitudinal interventions that examine the impacts on health outcomes (oral and general) by enhancing social support.

## 5. Conclusions

This study highlighted significant relationships between social support, socioeconomic factors, behavioral clustering, and oral health outcomes among older adults. Greater social support and networks were linked to healthier behaviors, though the effects on edentulism were shaped by broader influences like education and wealth. These findings underscore the need for future longitudinal research and for socially inclusive policies that address the social determinants of oral health to reduce inequalities and promote long-term well-being in aging populations.

## Figures and Tables

**Table 1:** Descriptive statistics of the sample.

Variables		Included (*n* = 2287)	Excluded (*n* = 2122)	*p*-Value ^a^
		%, Mean	%, Mean	
Gender				
	Male	49%	48%	
	Female	51%	52%	0.295
Ethnicity				
	White	99%	98%	
	Multi-ethnic	1%	2%	<0.001
Qualification				
	Less than O-level	26%	44%	
	O-level	30%	24%	
	Higher than A-level	44%	31%	<0.001
Age		62.2	66.9	<0.001
Wealth		3.6	3.1	<0.001
Social support		45.1	43.9	0.027
Social network		9.7	9.0	<0.001
Age (Wave 5)		66.3	71.2	<0.001
Edentulism (Wave 5)				
	Yes	8%	18%	
	No	92%	82%	<0.001
Smoking (Wave 7)				
	Non-smoker	89%	85%	
	Smoker	11%	15%	0.263
Alcohol intake (Wave 7)				
	14 or less unit per week	87%	91%	
	More than 14 units per week	13%	9%	0.385
Fruit and vegetable consumption (Wave 7)				
	Less than 5 portions per day	83%	81%	
	5 portion or more per day	17%	19%	0.401
Physical activity (Wave 7)				
	No or low activity per week	17%	41%	
	Moderate or high activity per week	83%	59%	<0.001

^a^ *p*-value from Chi-square test and *t*-test.

**Table 2:** Latent class probabilities and class descriptives.

Two Class Model		Class 1 (Risky) 7% (*n* = 137)	Class 2 (Healthy) 93% (*n* = 1910)
Item-response Probabilities			
Fruits and vegetables			
	Less than 5 portions per day	98%	81%
	5 portions or more per day	2%	18%
Smoking			
	Non-smokers	34%	98%
	Smokers	66%	2%
Physical activity			
	No or low activity per week	32%	16%
	Moderate or high activity per week	68%	84%
Alcohol intake			
	14 or less units per week	80%	87%
	More than 14 units per week	20%	13%
Class Descriptive Percentages			
Gender			
	Male	52%	50%
	Female	48%	50%
Education			
	Less than O-level	33%	26%
	O-level	40%	30%
	Higher than A-level	27%	44%
Edentulism			
	Yes	13%	7%
	No	87%	93%
Ethnicity			
	White	99%	99%
	Multi-ethnic	1%	1%
Class Descriptive Mean			
Age		59.3	62.4
Wealth		2.7	3.7
Social support		42.2	45.5
Social network		8.9	9.8

**Table 3:** SEM pathway for the association between social support and network and cluster of behaviors (Wave 3 to Wave 7) (*n* = 2287).

Variables		SC	95%CI	*p*-Value
Confirmatory factor analysis				
	Social support	0.61	(0.423, 0.800)	<0.001
	Social network	0.75	(0.517, 0.988)	<0.001
Direct effect to Edentulism				
	Social support and network	0.08	(0.025, 0.130)	0.004
Direct effect to Cluster of behaviors				
	Social support and network	0.15	(0.054, 0.239)	0.002
	Edentulism	0.12	(0.056, 0.182)	<0.001
	Cluster of behaviors Wave 3	0.23	(0.190, 0.280)	<0.001
	Gender	0.02	(−0.054, 0.098)	0.576
	Ethnicity	0.08	(−0.118, 0.277)	0.431
	Age at Wave 3	−0.40	(−1.705, 0.898)	0.544
	Age at Wave 5	0.62	(−0.686, 1.929)	0.352
Indirect effect to Cluster of behaviors (through edentulism)				
	Social support and network	0.01	(0.001, 0.017)	0.020
Total effect to Cluster of behaviors (direct + indirect)				
	Social support and network	0.16	(0.064, 0.0248)	0.001
Model fit				
	RMSEA	0.03	(0.017, 0.037)	
	CFI	0.95		
	TLI	0.91		

SC, standardized confection; RMSEA, root mean square error of approximation; CFI, comparative fit index; TLI, Tucker-Lewis index.

**Table 4:** SEM pathway for the association between social support and network and cluster of behaviors accounting for education and wealth (Wave 3 to Wave 7) (*n* = 2287).

Variables		SC	95%CI	*p*-Value
Confirmatory factor analysis				
	Social support	0.76	(0.606, 0.919)	<0.001
	Social network	0.61	(0.479, 0.737)	<0.001
Direct effect to social support and network				
	Wealth	0.14	(0.086, 0.195)	<0.001
Direct effect to Edentulism				
	Social support and network	0.05	(0.000, 0.101)	0.052
	Wealth	0.16	(0.114, 0.205)	<0.001
Direct effect to Cluster of behaviors				
	Social support and network	0.15	(0.042, 0.223)	0.004
	Edentulism	0.01	(0.021, 0.142)	0.008
	Cluster of behaviors Wave 3	0.19	(0.151, 0.240)	<0.001
	Gender	0.04	(−0.036, 0.119)	0.295
	Ethnicity	0.05	(−0.105, 0.212)	0.508
	Age at Wave 3	−0.25	(−1.535, 1.037)	0.700
	Age at Wave 5	0.46	(−0.830, 1.760)	0.482
	Education	0.06	(−0.024, 0.152)	0.155
	Wealth	0.21	(0.129, 0.286)	<0.001
Indirect effect to Cluster of behaviors (through edentulism)				
	Social support and network	0.01	(−0.001, 0.009)	0.112
Total effect to Cluster of behaviors (direct + indirect)				
	Social support and network	0.14	(0.046, 0.227)	0.003
Model fit				
	RMSEA	0.02	(0.014, 0.032)	
	CFI	0.96		
	TLI	0.93		

SC, standardized confection; RMSEA, root mean square error of approximation; CFI, comparative fit index; TLI, Tucker-Lewis index.

## Data Availability

The data used in this study are publicly available from the English Longitudinal Study of Ageing (ELSA) via the UK Data Service (https://ukdataservice.ac.uk/ (accessed on 31 August 2025)). Access to the dataset requires registration and is subject to the UK Data Service’s data sharing policy.

## Supplementary Materials

The following supporting information can be downloaded at: https://www.mdpi.com/article/10.3390/geriatrics10060142/s1, Figure S1: A flow chart of the study sample; Table S1: Latent class analysis model fit indices for behaviors at Wave 7; Table S2: Sensitivity analysis for the association between social support and network and fruit and vegetable consumption; Table S3: The interaction between socioeconomic factors and social support and network.
