# Supplementary material for: The Longitudinal Association Between Social Factors, Edentulism, and Cluster of Behaviors
Source: Geriatrics (Basel). 2025 Oct 31;10(6):142. doi: 10.3390/geriatrics10060142 (PMC12641908; doi:10.3390/geriatrics10060142)
Supplement: Supplementary file 1 [file geriatrics-10-00142-s001.zip › geriatrics-3913047-supplementary.pdf]

The Longitudinal Association between Social Factors, Edentulism and Cluster of Behaviors

Supplementary Material

Supplementary Figure S1 – A flow chart of the study sample.

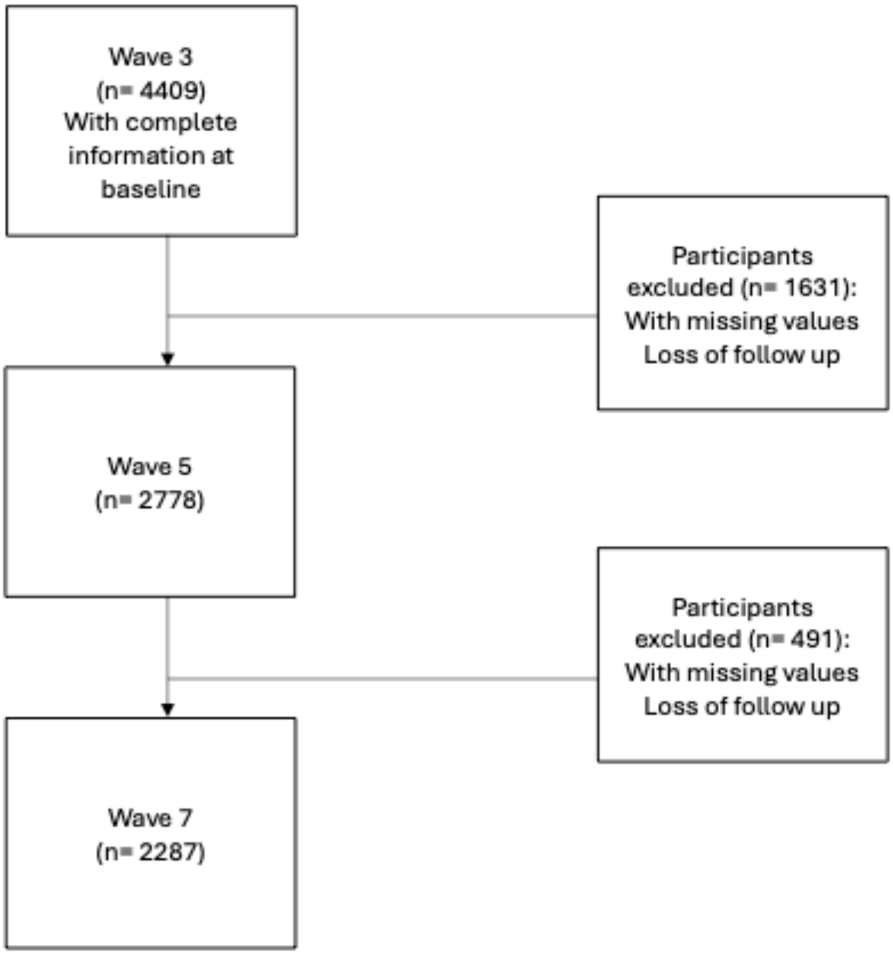

Supplementary Table S1 - Latent class analysis model fit indices for behaviors at Wave 7.

Class model	FB	AIC	BIC	A-BIC	Entropy
1 Class model	4	7223.96	7246.90	7234.19	-
2 Class model	9	7204.79	7256.41	7227.81	1.000
3 Class model	14	7203.17	7283.46	7238.98	0.681
4 Class model	19	7211.98	7320.95	7260.58	0.795

FP, free parameter; AIC, Akaike Information Criterion; BIC, Bayesian Information Criterion; A-BIC, Adjusted Bayesian Information Criterion.

Supplementary Table S2 – Sensitivity analysis for the association between social support and network and fruit and vegetable consumption (Wave 3 to Wave7) (n = 2287).

Variables	SC	95%CI	P-value
Confirmatory factor analysis			
Social support	0.76	(0.606, 0.919)	<0.001
Social network	0.61	(0.479, 0.737)	<0.001
Direct effect to social support and network			
Wealth	0.15	(0.086, 0.195)	<0.001
Direct effect to Edentulism			
Social support and network	0.05	(0.000, 0.101)	0.051
Wealth	0.16	(0.114, 0.205)	<0.001
Direct effect to Fruit and vegetable			
Social support and network	0.08	(0.042, 0.223)	0.040
Edentulism	0.02	(0.021, 0.142)	0.485
Fruit and vegetable Wave 3	0.18	(0.151, 0.240)	<0.001
Gender	0.04	(-0.036, 0.119)	0.203
Ethnicity	-0.01	(-0.105, 0.212)	0.702
Age at Wave 3	-0.29	(-1.535, 1.037)	0.538
Age at Wave 5	0.28	(-0.830, 1.760)	0.550
Education	-0.02	(-0.024, 0.152)	0.480
Wealth	0.01	(0.129, 0.286)	0.668
Indirect effect to Fruit and vegetable (through edentulism)			
Social support and network	0.01	(-0.001, 0.009)	0.496
Total effect to Fruit and vegetable (direct + indirect)			
Social support and network	0.08	(0.046, 0.227)	0.038
Model fit			
RMSEA	0.02	(0.014, 0.033)	
CFI	0.95		
TLI	0.92		

RMSEA, root mean square error of approximation; CFI, comparative fit index; TLI, Tucker–Lewis index.

Supplementary Table S3 - The interactions between socioeconomic factors and social support and network.

**Model**			
**Variables**	**OR**	**95% CI**	**P-value**
Social support	0.97	(0.92, 1.01)	0.172
Wealth	0.44	(0.21, 0.89)	0.023
Education	2.47	(0.91, 6.70)	0.076
Wealth* Social support	1.03	(1.01, 1.04)	0.001
Education* Social support	0.98	(0.96, 1.01)	0.126
Tooth loss at wave 5	1.99	(1.16, 3.42)	0.013
Cluster at Wave 3	11.35	(5.62, 22.90)	0.000
Sex	1.18	(0.84, 1.66)	0.335
Ethnicity	2.70	(0.59, 12.23)	0.197
Age at wave 3	0.95	(0.61, 1.48)	0.829
Age ta wave 5	1.11	(0.72, 1.73)	0.624
